# Less is more: downsampling x-ray images improves pose estimation accuracy

**DOI:** 10.1088/1873-4030/ae2909

**Published:** 2026-01-20

**Authors:** David E Williams, Michael J Rainbow, Dajung Yoon, Joseph J Crisco, Lauren Welte

**Affiliations:** 1 Musculoskeletal Biomechanics Research Facility, School of Engineering, Cardiff University, Cardiff, United Kingdom; 2 Department of Mechanical and Materials Engineering, Queen’s University, Kingston, Canada; 3 Department of Orthopaedics and Biomedical Engineering, Brown University, Providence, RI, United States of America; 4 Department of Mechanical Engineering, Faculty of Engineering, College of Natural and Applied Sciences, University of Alberta, Edmonton, Canada; 5 Department of Biomedical Engineering, Faculty of Engineering, College of Natural and Applied Sciences, University of Alberta, Edmonton, Canada

**Keywords:** pose estimation, fluoroscopy, image resolution, pose accuracy, motion analysis, tracking error, ankle

## Abstract

Biplanar videoradiography (BVR) is a gold-standard technique for quantifying *in vivo* bone motion, yet the influence of x-ray image resolution on pose estimation accuracy remains unexplored. This study investigates how downsampling x-ray images impacts model-based pose estimation, using high-speed BVR data from a participant with implanted tantalum beads. Images were downsampled from 2048 × 2048 to 512 × 512 using bicubic and nearest-neighbour interpolation. Across multiple bones and varying perturbation levels, downsampling significantly reduced rotational and translational errors when compared to full-resolution images for both interpolation results. Bicubic interpolation led to slightly improved pose accuracy for certain bones, demonstrating enhanced edge clarity that benefits the optimisation algorithm. Pose estimates for full-resolution images exhibited more outliers and greater variability for all the bones investigated. These findings highlight that downsampling images improves pose estimation accuracy even for challenging anatomical areas such as the ankle. We recommend bicubic downsampling to 512 × 512 pixels as a best practice for BVR tracking of the ankle complex, when using both automated optimisation and manual workflows.

## 1. Introduction

BVR is a powerful imaging modality for measuring three-dimensional (3D) bone and joint motion during dynamic tasks. By capturing dynamic x-ray images from two spatially calibrated cameras, BVR provides highly accurate measurements of an individual’s bone movements, including bones that are difficult or impossible to track externally, such as the scapula, patella, and talus. Reported accuracies range from 0.3 to 2.0*°* and 0.3–1.3 mm, depending on the protocol and joint system (Wang *et al* 2015, Iaquinto *et al* 2018, Pitcairn *et al* 2018, Akhbari *et al* 2019, Kage *et al* 2020, Setliff and Anderst 2024).

To estimate a bone’s position and orientation (pose) from BVR images, a 3D bone model is aligned with the x-ray data at each frame through a semi-automatic matching process (Akhbari *et al* 2019, Welte *et al* 2022). First, a partial volume is derived from a CT scan, containing only the bone of interest. The partial volume is virtually placed within the BVR volume and virtual x-rays are passed through it to create DRRs. The DRRs are projected onto the two calibrated x-ray images. The partial volume is manually rotated and translated until the DRRs visually match the x-ray images, providing an initial bone pose. Then, an optimisation fine-tunes the pose, minimising the normalised cross-correlation values between the DRRs and x-ray images (Nelder and Mead 1965, Kennedy and Eberhart 1995). This workflow is influenced by multiple factors, including input data quality and operator dependence, making it susceptible to error.

One potential source of error in the pose estimation process is the resolution of the x-ray images. The x-ray image sequences that capture dynamic tasks are acquired with high-speed video cameras. Modern cameras typically have a high resolution on the order of 2048 × 2048 pixels. Image processing filters are applied to both the x-ray images and the DRRs to enhance contrast and sharpen the edges of the bones. While it may seem intuitive that using as much information as possible (e.g. all 2048 × 2048 pixels), high-resolution images may have higher noise compared to lower-resolution images (Gonzalez and Woods 2017). Since the matching process relies on strong edges, its accuracy could vary with image resolution. While some researchers address this by downsampling their images before the matching process, the effects of image resolution on pose estimation accuracy have not been previously assessed. Understanding how image resolution affects accuracy will allow researchers to establish best practices for image processing prior to pose estimation.

This study evaluates how image resolution affects pose estimation accuracy, leveraging a rare dataset in which the participant had three or more metal beads surgically implanted into their ankle bones. These beads serve as precise markers, allowing for gold standard tracking of bone motion. Using this, the estimated pose’s accuracy can be evaluated. Since the pose estimation algorithm relies heavily on detecting strong edges, and downsampling can enhance edge contrast, we expected that lower-resolution images would provide higher accuracy during the matching process compared to high-resolution images.

## 2. Methods

An open-source BVR dataset was used as the gold standard for dynamic pose measurement (Welte *et al* 2022). In summary, the data set consists of a single participant (M, 49 years, 83 kg, 1.75 m) who had three or more 1 mm tantalum beads implanted into their calcaneus (3 beads), talus (4 beads) and tibia (5 beads). CT images were captured (Resolution: 0.441 mm × 0.441 mm × 0.625) and segmented to generate bone surface models and partial volumes of the individual’s right calcaneus, talus and tibia. During the experimental protocol, the participant hopped to a metronome at 108 bpm while BVR captured two x-ray image pairs at 250 Hz. A total of 35 frames were included in the analysis, from when the heel contacts the floor, to the frame where at least one bone is out of view. The reference bone poses were determined by digitising bead positions in XMALab (Brown University, https://bitbucket.org/xromm/xmalab/src/master/), which is the gold standard for pose estimation (Brainerd *et al* 2010, Miranda *et al* 2011, Knörlein *et al* 2016). The tantalum beads in the x-ray images were then digitally removed to avoid biasing the model-based pose estimation algorithm.

**Figure 1.**
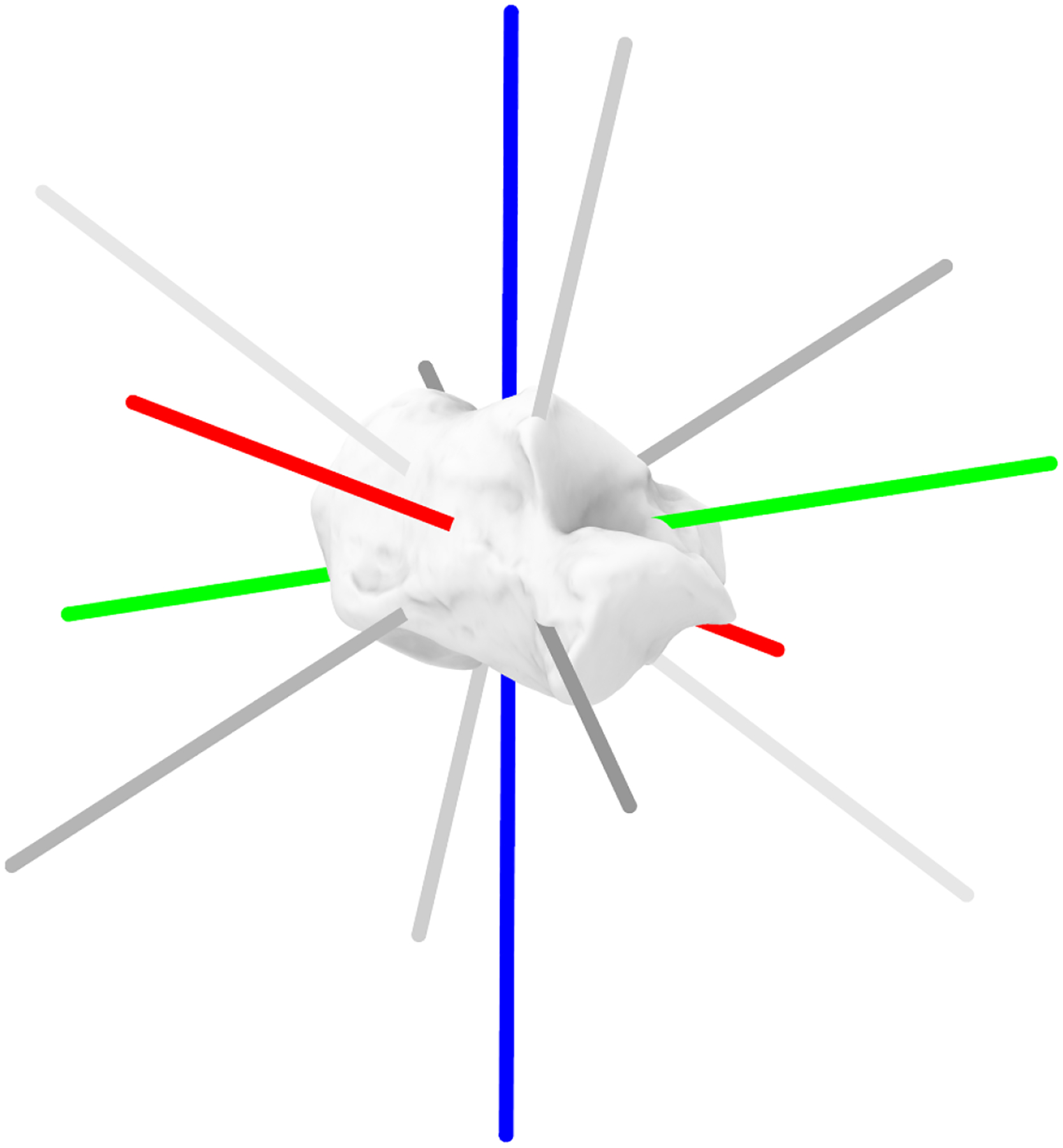
Shows the applied inertial coordinate system to the calcaneus with *X, Y* and *Z* represented as red, green and blue, respectively. The diagonal unit vectors are shown in grey.

To simulate manual initial seed poses, the gold standard bead-tracked poses were perturbed. First, each bone’s inertial coordinate system was defined, with the origin set at its inertial centre. Rotational perturbations of varying magnitudes (±2*°*, ±4*°*, and ±8*°*) were applied individually about the bone’s *X, Y*, and *Z* axes, as well as about four additional axes formed by diagonal unit vectors (figure 1). These perturbations represented different levels of initial bone pose accuracy. For each bone, this process generated 42 perturbed poses (seven axes with both positive and negative rotational perturbations applied).

BVR images were downsampled from the original capture image resolution, 2048 × 2048, to 512 × 512 using two different approaches: bicubic and nearest-neighbour interpolation. Bicubic interpolation uses a weighted average of 4 × 4 grids (the nearest 16 pixels) around the output pixel (Keys 1981). To test performance against filtering effects, and to provide a comparison against a non-filtered method, we also included nearest-neighbour interpolation, which was applied without any adjustment for anti-aliasing, thereby avoiding the introduction of additional filtering. Nearest-neighbour interpolation assigns each output pixel in the 512 × 512 image the values of the closest corresponding pixel in the original 2048 × 2048 image. This method considers only the nearest pixel and does not introduce any new pixel values (Parker *et al* 1983). Bicubic produces a smoothed image compared with the sharper image from nearest neighbour interpolation. This was performed for both approaches using the in-built imresize function in MATLAB (The Mathworks, Inc.)

An open-source image-based 2D–3D motion tracking software, SlicerAutoscoperM (SA^M^, Brown University, https://autoscoperm.slicer.org/) estimated the bone poses. This software supports batch processing through a transmission control protocol socket in MATLAB, enabling high-throughput pose estimation. For each frame, the bone’s perturbed pose was refined using a particle swarm optimisation (Kennedy and Eberhart 1995). This optimisation fine-tunes the individual bone poses until the normalised cross-correlation values between the DRRs and x-ray images are minimised. These newly optimised pose estimates were exported for each perturbation across all three bones using each image resolution (fullres, bicubic, nearest neighbour). At each frame, the difference between the gold standard bead tracking pose and the perturbed and re-optimised pose was quantified using the helical axis of motion parameters. To simplify the analysis, we defined rotational error as the helical axis angle, phi, that describes how much rotation occurs about the helical axis. Translational errors were quantified as the translation that occurs along the axis (Panjabi *et al* 1982).

For each image resolution and perturbation (±2*°*, ±4*°*, and ±8*°*), the variations in helical rotation and translation were combined across frames. To assess variability and accuracy across perturbations and resolutions, the cumulative error, median, LOA and MAD were calculated for each dataset. Cumulative error, representing the sum of absolute differences from the gold standard, provided an overall measure of deviation. The median and LOA (calculated as the median ±1.45× interquartile range of the differences, with any negative lower bounds truncated to 0 as the data represent absolute errors) were used to evaluate central tendency and spread, while the MAD, a robust measure unaffected by outliers, allowed for comparison of variability across resolutions. Outliers were identified using the Hampel identifier, which calculates deviations from the median relative to MAD, with a threshold of 3.5 (Hampel 1974, Wilcox 2012).

Statistical analyses assessed differences in helical rotation and translation across image resolutions separately for each combination of bone and perturbation level. Kruskal–Wallis tests were conducted to determine if significant differences existed among image resolutions within each perturbation level and bone. For cases where the Kruskal–Wallis test was significant (*p* < 0.05), Dunn’s post-hoc test with Bonferroni correction was applied to identify specific pairwise differences. All statistical analyses were performed using R v4.3.2 (R Core Team n.d.).

## 3. Results

The downsampled images significantly outperformed the full resolution images for all comparisons except calcaneus rotation at ±2*°*, with lower median differences and smaller LOA for both helical rotation and translation (tables 1–3). At 2*°* of perturbation, which approximates a careful initial guess, full-resolution helical rotation median differences [LOA] ranged from 0.3*°* [2.73*°*] to 1.2*°* [155.38*°*] across the three bones, compared to much lower values (0.1*°* [0.3*°*] to 0.6*°* [0.9*°*]) for downsampled images using the nearest neighbour method. At 8*°*, which approximates a rough initial guess, this difference was even more pronounced: full-resolution median differences [LOA] ranged from 3.4*°* [10.22*°*] to 105.4*°* [252.5*°*], while nearest neighbour downsampling maintained significantly lower values (0.1*°* [0.4*°*] to 0.6*°* ([1.4*°*]). LOA values above 180*°* reflect the calculation rather than the underlying measurements. The same trend exists for helical translation, with downsampled images consistently yielding smaller median differences, LOA, cumulative errors, and MADs across all bones and perturbation levels.

When comparing the two downsampling methods directly, the nearest neighbour approach generally had higher median differences, LOA, and MAD values compared to bicubic interpolation. For both the talus and calcaneus, small but statistically significant differences between the two downsampling methods suggest that bicubic interpolation generally outperforms the nearest neighbour algorithm.

The full resolution images had the most outliers as defined by the Hampel identifier, typically caused by frames where the pose estimation algorithm failed to reach a solution. No outliers were identified for the calcaneus rotation at ±4*°* and ±8*°* using full resolution images (table 1). This occurred because the overall median and MAD were high enough that large errors were not flagged as outliers. Visual inspection confirmed that the full resolution images generally resulted in poor calcaneus pose estimations compared to downsampled images.

## 4. Discussion

This study aimed to evaluate the effects of image resolution and initial guess accuracy on pose-estimation accuracy, using gold-standard *in vivo* BVR data of the calcaneus, talus and tibia. Overall, downsampling using either bicubic or nearest-neighbour algorithms provided clear improvements in accuracy over full-resolution images across all perturbations and bones. Although the differences between the downsampling approaches were much more subtle, there were no instances where the nearest-neighbour algorithm outperformed the bicubic algorithm. We therefore recommend that users downsample their images using the bicubic algorithm to improve pose-estimation accuracy when processing BVR data.

**Figure 2.**
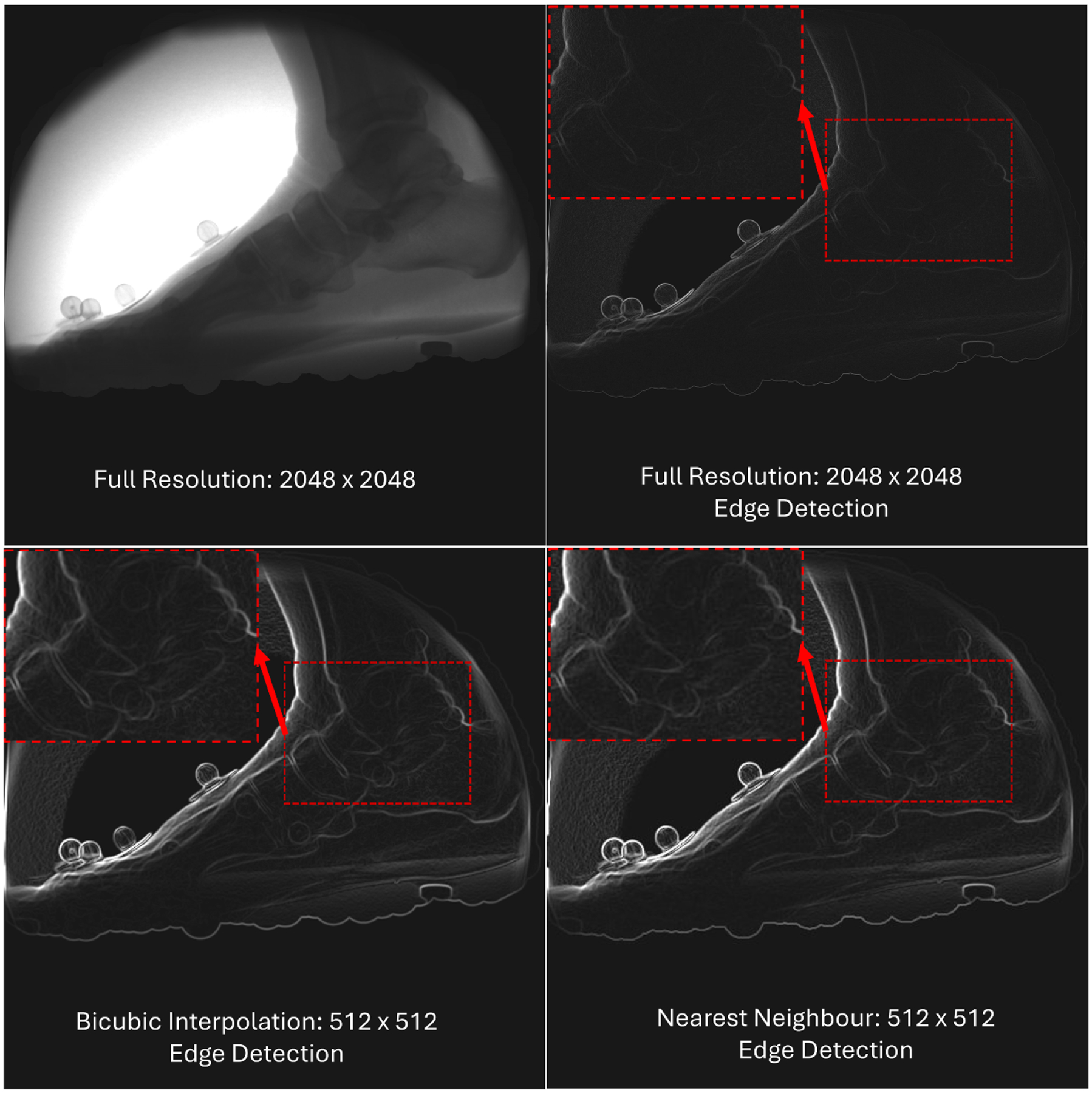
X-ray of foot during activity at different resolutions and edge detection applied. The bottom images show the downsampled images have stronger lines compared with the full resolution (top-right).

This result likely arises as the alignment of DRRs to the 2D biplanar x-ray images relies heavily on edge detection algorithms, such as the Sobel edge detection algorithm used by SA^M^. In high-resolution images, the edges of bone structures often appear finer and less prominent due to the increased pixel density, which captures subtle transitions in anatomical detail. In contrast, downsampling combines adjacent pixels, effectively enhancing the contrast and definition of key anatomical features. This process results in thicker, more distinct edges, which improves their detectability and facilitates the performance of pose estimation algorithms (figure 2).

These enhanced edges may also be of particular benefit when aligning with CT-derived DRRs, as their resolution is often more comparable to the downsampled images (typically 512 × 512 pixels per slice). Additionally, downsampling reduces the data size of input images which could benefit certain pose estimation algorithms’ computational efficiency.

Pose estimation performance improved for certain bones when comparing the two downsampling methods. Bicubic interpolation produces smoother images by taking a weighted average of multiple pixels during downsampling. This helps preserve more of the original information and creates a gradual gradient at bone edges. In contrast, nearest-neighbour interpolation selects a single pixel’s value, which can introduce aliasing errors, leading to jagged edges (Parsania and Pv 2016). Further research is needed to determine whether other interpolation methods could further enhance pose estimation accuracy.

The effects of downsampling were particularly evident for the tibia and talus bones, where high-resolution images led to significant challenges in pose estimation accuracy. The tibia produced many outliers (Helical rotation: 143; translation: 125 at ±2 perturbation), especially during phases of the motion where most of the diaphysis/metaphysis was out of the field of view. For the BVR imaging, this occurred at the start and end of the movement. Downsampling was able to provide strong enough features for the distal end of the tibia to overcome these challenges for the nearest neighbour (29 and 39 outliers at the ±2*°* perturbation for helical rotation and translation respectively) and bicubic methods (34 and 38 outliers at the ±2*°* perturbation for helical rotation and translation respectively). At higher perturbations, the number of outliers increased for both downsampling methods but decreased for full-resolution images. This occurred because outlier detection depends on the MAD which remained consistent (~0.05*°* rotation, ~0.45 mm translation) across perturbation levels for downsampled images, but increased substantially (from 0.20*°*/0.96 mm to 2.00*°*/10.33 mm) at the 8*°* perturbation for full resolution images. As a result, fewer values were identified as outliers at higher perturbations for the full-resolution images.

Similarly, the talus posed considerable challenges for high-resolution images, likely due to its anatomical location. The talus is enclosed by adjacent bones, and this bone overlap makes edge detection particularly challenging, resulting in many outliers throughout the movement (helical rotation: 218; translation: 199 outliers at ±2*°* perturbation). Downsampling substantially improved talus tracking by enhancing edge clarity and reducing interference from surrounding bone structures. Bicubic downsampling performed the best, with only 32 (rotation) and 37 (translation) outliers across all perturbations.

In contrast, the calcaneus, being less obstructed by surrounding bones, demonstrated fewer tracking issues (outliers) across resolutions. However, it consistently had higher median difference and levels of agreement when compared to the other bones. This is most likely due to the posterior edge of the bone being slightly out of the field of view for one of the x-ray views, making it challenging for all three methods accurately estimate the pose. Despite this for 4*°* and 8*°* perturbation the median difference, LOA and cumulative error were all significantly reduced for the two downsampling methods. This underscores the critical importance of clear bone visibility in the x-ray images for accurate pose estimation.

It is important to note that this data is based on a single volunteer performing one activity captured in 35 frames. However, a validation study on the ankle with a notably larger dataset (999 frames) reported similar accuracy when applying downsampling techniques, with mean error (bias) <0.5*°* (1.8*°*) and <0.8 mm (3.1 mm) (Morton *et al* 2025). These findings are consistent with accuracy ranges reported in the wider literature (0.3–1.3 mm and 0.9–3.3*°*) (Setliff and Anderst 2024). As shown by the improved performance of bicubic over nearest neighbour, filtering of the x-ray images prior to pose estimation will likely also have a direct impact on the accuracy of the data. Filtering and downsampling images will most likely be the best combination for accurate pose estimation. For the purposes of this study, and to avoid introducing bias through manual corrections, the algorithm was allowed to perform the matching autonomously, with the understanding that this automated method is not truly representative of how a user would typically carry out the pose estimation process. This is evident by some of the more extreme outliers. The recommended approach involves the user manually matching key frames positioned throughout the activity, followed by using particle swarm optimisation to refine the remaining frames. As there are currently no fully automated algorithms for tracking ankle bones, the algorithm occasionally produces erroneous results. In practice, the user would remove these errors and rely on surrounding frames and spline curve fitting of the poses to correctly position the bone. The goal of achieving fully automated pose estimation for the ankle may be more attainable with the use of downsampled images.

The ideal level of downsampling will likely depend on factors such as bone anatomy, the nature of the activity being captured, and the geometry of the BVR system. While this study focused on a single resolution (512 × 512), future work should explore the effects of both higher and lower resolutions (e.g. 1024 × 1024 or 256 × 256). There is likely a lower bound below which resolution becomes too coarse to retain sufficient edge detail, and an upper bound where noise and weak edges begin to degrade pose estimation performance. As demonstrated in this study, the method of downsampling also plays a key role. Bicubic interpolation, in addition to reducing resolution, effectively smooths the image by averaging adjacent pixels, which acts as a form of low-pass filtering. This smoothing may enhance edge continuity and reduce high-frequency noise, contributing to its improved performance over nearest-neighbour interpolation. The interaction between this inherent filtering effect and any additional preprocessing steps—such as edge sharpening or contrast adjustment—may further influence accuracy. A more comprehensive understanding of how resolution, downsampling method, and filtering interact will be important for establishing best practices in BVR image processing across different anatomical regions and movement tasks.

Downsampling plays an important role in enhancing the accuracy and efficiency of pose estimation algorithms for BVR data of the ankle. Based on these findings, we recommend continuing to collect high-resolution data, as this may prove valuable for future research questions. However, when applying the particle swarm algorithm or other manual or automated techniques, we recommend downsampling images to 512 × 512 using the bicubic method.

## Figures and Tables

**Table 1.** Descriptive statistics for helical rotation differences between full resolution and downsampled images.

	Tibia											
Perturbation	±2*°*				±4°				±8*°*			
	Rotation				Rotation				Rotation			
Helical difference	Median (LOA*)/°*	Cumulative error/°	MAD*/°*	No. of outliers	Median (LOA)/°	Cumulative error/°	MAD*/°*	No. of outliers	Median (LOA)/°	Cumulative error*/°*	MAD*/°*	No. of outliers
Fullres	0.28 (2.73)	1451.30	0.20	143	1.57 (7.04)	2787.84	1.43	70	3.38 (10.22)	4842.40	2.00	101
Bicubic	0.10 (0.27)	62.19	0.06	34	0.10 (0.26)	71.34	0.05	49	0.10 (0.29)	152.19	0.06	55
Nearest neighbour	0.10 (0.26)	60.38	0.05	29	0.10 (0.26)	75.20	0.05	46	0.12 (0.31)	255.11	0.06	67
	Talus											
Perturbation	±2*°*				±4°				±8*°*			
	Rotation				Rotation				Rotation			
Helical difference	Median (LOA*)/°*	Cumulative error/°	MAD*/°*	No. of outliers	Median (LOA)/°	Cumulative error/°	MAD*/°*	No. of outliers	Median (LOA)/°	Cumulative error*/°*	MAD*/°*	No. of outliers
Fullres	1.18 (155.38)	23 056.76	1.12	218	105.08 (259.24)	35 558.38	3.97	178	105.40 (252.53)	38 215.37	3.59	155
Bicubic	0.15 (0.39)	88.13	0.08	16	0.15 (0.42)	97.41	0.09	17	0.14 (0.41)	101.89	0.08	32
Nearest neighbour	0.16 (0.51)	119.40	0.11	23	0.17 (0.53)	140.25	0.11	48	0.17 (0.57)	164.71	0.12	49
	Calcaneus											
Perturbation	±2*°*				±4°				±8*°*			
	Rotation				Rotation				Rotation			
Helical difference	Median (LOA*)/°*	Cumulative error/°	MAD*/°*	No. of outliers	Median (LOA)/°	Cumulative error/°	MAD*/°*	No. of outliers	Median (LOA)/°	Cumulative error*/°*	MAD*/°*	No. of outliers
Fullres	0.51 (9.98)	1720.44	0.38	141	5.47 (20.88)	3047.00	5.01	0^a^	7.44 (22.39)	3627.32	5.70	0^a^
Bicubic	0.50 (0.83)	267.23	0.15	16	0.54 (0.88)	279.78	0.15	3	0.58 (1.11)	375.22	0.20	22
Nearest neighbour	0.55 (0.93)	280.69	0.16	2	0.58 (1.03)	298.58	0.18	11	0.62 (1.32)	447.41	0.24	28

a No outliers were detected as the Hampel identifier considers variability (MAD), because of the high median and MAD values, all data points remained within the threshold.

**Table 2.** Descriptive statistics for helical translation differences between full resolution and downsampled images.

	Tibia											
Perturbation	±2*°*				±4*°*				±8*°*			
	Translation				Translation				Translation			
Helical difference	Median (LOA)/mm	Cumulative error/mm	MAD/mm	No. of outliers	Median (LOA)/mm	Cumulative error/mm	MAD/mm	No. of outliers	Median (LOA)/mm	Cumulative error/mm	MAD/mm	No. of outliers
Fullres	1.42 (6.92)	3405.56	0.96	125	5.39 (28.80)	6545.08	4.84	93	15.66 (48.05)	11 098.27	10.33	28
Bicubic	0.61 (1.94)	453.51	0.44	38	0.64 (1.91)	482.88	0.42	35	0.68 (2.16)	767.27	0.47	65
Nearest Neighbour	0.71 (1.74)	440.46	0.36	39	0.68 (1.91)	458.58	0.42	35	0.74 (2.73)	1308.86	0.45	88
	Talus											
Perturbation	±2*°*				±4°				±8*°*			
	Translation				Translation				Translation			
Helical difference	Median (LOA)/mm	Cumulative error/mm	MAD/mm	No. of outliers	Median (LOA)/mm	Cumulative error/mm	MAD/mm	No. of outliers	Median (LOA)/mm	Cumulative error/mm	MAD/mm	No. of outliers
Fullres	5.59 (44.42)	7298.15	5.36	199	26.25 (61.01)	12 134.28	5.99	115	27.70 (26.95)	16 719.88	4.90	94
Bicubic	0.76 (2.32)	480.77	0.53	33	0.75 (2.30)	494.29	0.52	37	0.78 (2.37)	501.85	0.52	37
Nearest Neighbour	0.94 (2.78)	677.45	0.59	58	1.01 (2.94)	676.71	0.64	51	1.02 (3.08)	777.46	0.64	57
	Calcaneus											
Perturbation	±2*°*				±4°				±8*°*			
	Translation				Translation				Translation			
Helical difference	Median (LOA)/mm	Cumulative error/mm	MAD/mm	No. of outliers	Median (LOA)/mm	Cumulative error/mm	MAD/mm	No. of outliers	Median (LOA)/mm	Cumulative error/mm	MAD/mm	No. of outliers
Fullres	3.32 (9.73)	2703.40	1.66	91	6.70 (23.10)	4315.65	6.70	1	10.32 (26.26)	5243.31	5.60	3
Bicubic	0.57 (1.57)	371.95	0.34	29	0.70 (1.83)	458.40	0.70	33	0.82 (2.43)	880.89	0.47	59
Nearest Neighbour	0.66 (1.83)	435.53	0.38	34	0.68 (2.22)	517.60	0.68	37	1.06 (3.25)	1118.72	0.68	54

**Table 3.** Kruskal–Wallis analysis results using Dunn’s post-hoc test with Bonferroni correction to test differences between full resolution, downsampling using bicubic and nearest neighbour interpolation.

	Tibia											
Perturbation	±2*°*				±4*°*				±8*°*			
Helical difference	Rotation		Translation		Rotation		Translation		Rotation		Translation	
	*Z*-score	Adjusted *p*-value	*Z*-score	Adjusted *p*-value	*Z*-score	Adjusted *p*-value	*Z*-score	Adjusted *p*-value	*Z*-score	Adjusted *p*-value	*Z*-score	Adjusted *p*-value
Fullres vs bicubic	**−15.18**	⩽**0.001**	**−12.78**	⩽**0.001**	**−20.62**	⩽**0.001**	**−20.67**	⩽**0.001**	**−24.89**	⩽**0.001**	**−25.14**	⩽**0.001**
Fullres vs NN	**15.14**	⩽**0.001**	**12.61**	⩽**0.001**	**20.03**	⩽**0.001**	**20.63**	⩽**0.001**	**22.80**	⩽**0.001**	**23.31**	⩽**0.001**
Bicubic vs NN	−0.04	1	−0.17	1	−0.60	1	−0.05	1	−2.09	0.110	−1.83	0.201
	Talus											
Perturbation	±2*°*				±4°				±8*°*			
	Rotation		Translation		Rotation		Translation		Rotation		Translation	
Helical difference	*Z*-score	Adjusted *p*-value	*Z*-score	Adjusted *p*-value	*Z*-score	Adjusted *p*-value	*Z*-score	Adjusted *p*-value	*Z*-score	Adjusted *p*-value	*Z*-score	Adjusted *p*-value
Fullres vs bicubic	**−20.70**	⩽**0.001**	**−18.26**	⩽**0.001**	**−23.77**	⩽**0.001**	**−25.46**	⩽**0.001**	**−26.03**	⩽**0.001**	**−27.73**	⩽**0.001**
Fullres vs NN	**18.29**	⩽**0.001**	**14.68**	⩽**0.001**	**21.97**	⩽**0.001**	**22.42**	⩽**0.001**	**24.09**	⩽**0.001**	**24.81**	⩽**0.001**
Bicubic vs NN	**−2.41**	**0.048**	**−3.58**	**0.001**	−1.80	0.216	**−3.04**	**0.007**	−1.95	0.154	**−2.92**	**0.011**
	Calcaneus											
Perturbation	±2*°*				±4°				±8*°*			
	Rotation		Translation		Rotation		Translation		Rotation		Translation	
Helical difference	*Z*-score	Adjusted *p*-value	*Z*-score	Adjusted *p*-value	*Z*-score	Adjusted *p*-value	*Z*-score	Adjusted *p*-value	*Z*-score	Adjusted *p*-value	*Z*-score	Adjusted *p*-value
Fullres vs bicubic	−0.70	1	**−24.02**	⩽**0.001**	**−13.62**	⩽**0.001**	**−23.34**	⩽**0.001**	**−21.88**	⩽**0.001**	**−22.13**	⩽**0.001**
Fullres vs NN	−1.07	0.856	**21.53**	⩽**0.001**	**12.09**	⩽**0.001**	**22.96**	⩽**0.001**	**19.94**	⩽**0.001**	**19.62**	⩽**0.001**
Bicubic vs NN	−1.77	0.229	**−2.49**	**0.039**	−1.52	0.385	−0.38	1	−1.94	0.157	**−2.51**	**0.036**

*Note*: Bold shows statistical significance below *P* < 0.05. Bold and underline shows statistical significance below *P* < 0.001.

## Abbreviations

BVR: Biplanar videoradiography
CT: Computed tomography
DRRs: Digitally reconstructed radiographs
LOA: Limits of agreement
MAD: Median absolute deviation
